# The Changeable Positioning of the Couch and Repositioning to Face-to-Face Arrangement in Psychoanalysis to Facilitate the Experience of Being Seen

**DOI:** 10.3389/fpsyg.2021.718319

**Published:** 2021-11-12

**Authors:** Bariş Önen Ünsalver, Alper Evrensel, Fatma Duygu Kaya Yertutanol, Aslihan Dönmez, Mehmet Emin Ceylan

**Affiliations:** ^1^Department of Psychiatry, School of Medicine, Uskudar University, Istanbul, Turkey; ^2^Department of Psychology, Bogaziçi University, Istanbul, Turkey

**Keywords:** gaze, eye contact, couch position, object relations, psychoanalysis

## Introduction

The main therapeutic relationship tools in the psychoanalytic process are transference, countertransference, and free association. Psychoanalytic space that constitutes the framework of the psychoanalytic method, the objects in the room, and especially the features such as the positioning of the couch or the chair, duration of each session, frequency of sessions, make it possible for these therapeutic tools to be activated. Although the couch or chair placement may change in different psychoanalytic schools, in classical psychoanalysis, the couch is traditionally positioned so that the analyst does not make eye contact with the analysand. This placement prevents eye-to-eye contact and facilitates free-association, and prepares the basis for the analysand to convey their free associations without feeling shame, fear, anxiety, or without the pressure of feeling these negatively valued feelings less (Adler and Bachant, 1996). However, some analysts oppose the idea of the couch as a facilitator of transference and free-association (Wolf, 1995) and some suggest using lying down and sitting up positions when necessary on a client-to-client basis (Celenza, 2005). Schachter and Kächele (2010) reported in their detailed review article that there are conflicting data regarding the necessity and validity of using the couch in psychoanalysis and that there is no empirical evidence to show that everyone from the psychoanalytic process should be seen on the couch. Current psychoanalysts and early analysts such as Erich Fromm and Harry Stuck Sullivan opposed both the psychoanalytic framework and the rigid stance on using the couch. They propose that it is possible to switch from the couch to a face-to-face sitting position with regard to the character of psychopathology, the practice of the psychoanalytic school pursued, or new situations that will arise here and now in the therapeutic process.

In this paper, the theory of change in psychoanalysis is accepted to be based on object relations approach. The goal of treatment is a change in the analysand's arrested or dysfunctional object relationship structure. It can be said that the essential and prominent component that establishes the therapeutic alliance is the mutual gaze between the client and the therapist. Eye contact sends a message to the receiver: “I am present with you.” In psychoanalytical relationship the analyst is expected “to be with” the analysand rather than “do with” the analysand (Ogden, 2004). When the analyst is sitting behind the couch preventing any eye contact, the analysand might not experience this self-understanding or receive any message via eyes of the analyst of them being there together. In this article we focus on eye gaze and the experience of being seen to support the proposition about changing the positions of the analyst and analysand when necessary.

## Eye-to-Eye Contact to Form the Object Relationship

Babies have a propensity to seek eye contact (Baron-Cohen, 1995). Especially in the first months, the babies monitor and manipulate the caregiver through eye movements and ensure that their needs are seen. The contrasting white of the sclera from the iris makes it easier for the mother to be alerted to this searching gaze by the baby and helps her follow the baby's gaze and become receptive and attuned to the baby's needs. There starts a proto-conversation via mutual gaze, as described by Schore (2012). In case of excessive stimulation from the caregiver, the baby regulates themselves by turning their gaze away (Stern, 1985). The first object relation is established through eye-to-eye contact and breaking of this contact, which occurs thousands of times.

The first special relationship between the mother and baby will form the template for future relationships (Schore, 2012). Therefore, it is accepted as a prototype for a therapeutic relationship (Gray, 1994). As a secure relationship develops in time, the baby is “held” and “contained” in the mother's gaze. According to Winnicott, the mother's “holding” confines both physical holding of the infant and the entire environmental provision for the infant. At first the mother holds and digests the infantile anxiety and reflects back to the infant with a modified affect that is conveyed mostly through the mother's gaze, keeping the infant from experiencing a sense of annihilation. Mother's gaze is a salient part of this holding environment (Winnicott, 2005). Bion states that the primary function of the mother in early infancy is to become a “container” for the frustration and pain of the infantile vulnerable ego (Meltzer et al., 2007). Mother's calm gaze can become a “container” of the baby's mind.

Based on this information, it seems necessary for the analysand, whose early attachment experiences surface through transference or who has regressed in a therapeutic environment such as psychoanalytic therapy, to re-establish “holding” and “containing” eye contact with the “new object” so that they will experience attunement in order to reconstruct and repair early object relations. Psychoanalysis may lay the groundwork for accelerating the recovery process with such simple interventions as coming into eye contact to help the analysand be seen and regulate themselves when the need arises. However, the analysand who does not see their analyst on the couch might miss this opportunity of reparation.

## Pupil Mimicry and the Experience of Being Seen

During eye contact, pupil sizes synchronize between partners, where the pupils of one party dilate or constrict in synchrony with the dilation and constriction of the pupils of the other party (Harrison et al., 2006). This is called pupil mimicry. It has been suggested that we understand information about the inner state of the other through unconscious and involuntary pupil mimicry (Kret et al., 2015). According to pupil mimicry studies, an arousal response is recorded in the amygdala when both pupils are dilated or a decreased arousal response in the amygdala when there is pupil constriction. Therefore, it is probable that when the therapist and the client face each other unconscious and involuntary pupil mimicry may occur, and the autonomic nervous systems of the client and the therapist may resonate through the mutual gaze. If the therapist has an insight into their bodily sensations and has available personal resources to manage their autonomic responses as the sensations emerge, then the therapist might intentionally relax themselves in situations where the client has overwhelming anxiety and help the client to regulate themselves through the therapist's pupils. The same pupillary response also serves to build trust between two people, which is thought to be built via oxytocin increase during pupil mimicry (Nagasawa et al., 2015). It might also be speculated that the experience of “being seen” by the therapist in real-time through eye-to-eye contact may also evoke the experience of “being held” through increasing oxytocin. This would be touching each other without actually touching. Having said all these about pupil mimicry, it must be said that most therapists don't probably actually see the client's pupils and further research is needed to measure whether there is pupillary mimicry among people sitting across each other during psychotherapy.

Experience of being seen by someone else might elicit affect-related psychophysiological responses in the person who receives the eye gaze (Hietanen et al., 2018). Studies have suggested that the gaze of a living person only initiates the effect of eye contact on the autonomic nervous system (Prinsen and Alaerts, 2019). These psychophysiological responses arise only during mutual and direct gaze where both gazers are in a live interaction (Prinsen and Alaerts, 2019). The perception of being seen or knowing that one is seen causes electrophysiological changes that can be measured with electroencephalography (EEG) electromyography (EMG) (Pönkanen et al., 2011; Myllyneva and Hietanen, 2015; Hietanen et al., 2018; Jarick and Bencic, 2019). These changes are in the form of arousal response determined by increased sympathetic activation in the autonomic nervous system, increased left frontal activity that can be associated with the motivation to get closer to the other, and EMG response associated with positive emotions (zygomaticus major activation and corrugator supercilii muscle relaxation) (Pönkanen et al., 2011; Hietanen et al., 2018). In a neutral context, those subjects who had the experience of being exposed to direct gaze had greater zygomaticus response and lesser corrugator activity than those exposed to averted gaze in a study by Hietanen et al. (2018), suggesting that provide evidence that, in a neutral context, another individual's direct gaze is an affiliative, positive signal.

These affect-related psychophysiological responses to the experience of being seen can be likened to the prior experience of being seen by the mother or any other object relation where the experience of “being seen” and “being accepted” helps the healthy development of self rather than the “false self” as described by Winnicott (2005).

In a recent study by Hietanen et al. (2020), 32 test subjects were exposed to three interaction conditions with the same model in the direct and averted gaze: live face-to-face, live video calls, and recorded videos. Skin conductance response was used as a measure of autonomic arousal, and facial EMG was used for facial reactions. The direct gaze caused more arousal than averted gaze, but not in the recorded video condition. On the other hand, EMG responses revealed more positive affective facial responses to direct gaze than averted gaze in all three conditions. This study suggests that knowing that someone is seen by someone in live interaction or a live video call increases autonomic arousal. The fact that direct gaze is associated with higher arousal underlines the importance of face-to-face interaction. So, when the analysand is lying down on the couch, not facing the analyst, they can still feel this autonomic arousal because they will know that the analyst is seeing them, but the arousal will be mild, which could help facilitate free association and introduction of shame-inducing thoughts.

Moving forward from the Hietanen et al. (2020) study, we suggest that it is not enough for the therapist and the client to be in the same room in order for the client to have a powerful “being seen” experience which would trigger neurobiological change but their coming into eye-contact is needed, whether in the same room or during an online therapy session. On the other hand the findings of the same study brings another point into attention: The experience of being seen increases arousal which might be experienced by the person being observed (the analysand) as anxiety provoking and might disturb the interaction between the analyst and analysand. This would then support the idea of switching back to the couch when the analysand's free-associations decrease or becomes overtly anxious when face-to-face. However, instead of switching to couch, turning the eyes away from the analysand for the rest of the session could also help the person regulate themselves.

## When to Reposition to Face-to-Face Arrangement

Clients with paranoid and borderline personality features and overwhelming anxiety may be suitable for a face-to-face arrangement. The “container” function of the gaze of the analyst might prevent further formation of psychotic thinking as described by Bion (Meltzer et al., 2007) in these clients. The attuned gaze can become the introjected good object in clients who commonly use projective identification. Changing into the face-to-face position could be appropriate at critical points during psychoanalysis where the analysand might show prolonged resistance reactions such as being silent or talking over things irrelevant to the process or skipping more sessions than that was agreed.

## Conclusion

Considering the essential neurobiological regulatory effects of mutual gaze between the mother and the infant on the developing baby, the experience of being seen by the therapist is expected to have significant neurobiological transformative effects on the client. We propose that, based on the neurobiological effects of mutual eye gaze, at critical points during the session or the working through of specific topics where the client needs to be seen, the analyst asks the client to transition from lying down on the couch without facing the psychoanalyst to a sitting position in a way that allows face-to-face mutual eye contact may have positive therapeutic effects.

## Author Contributions

BÖ formed the idea, did literature research, and wrote the manuscript. AE helped with the development of the idea and wrote the manuscript. FK did literature research and wrote the manuscript. AD helped with the development of the main idea. MC formed the idea during brainstorming sessions with BÖ. All authors contributed to the article and approved the submitted version.

## Conflict of Interest

The authors declare that the research was conducted in the absence of any commercial or financial relationships that could be construed as a potential conflict of interest.

## Publisher's Note

All claims expressed in this article are solely those of the authors and do not necessarily represent those of their affiliated organizations, or those of the publisher, the editors and the reviewers. Any product that may be evaluated in this article, or claim that may be made by its manufacturer, is not guaranteed or endorsed by the publisher.
